# Loss of H3K27me3 expression in canine nerve sheath tumors

**DOI:** 10.3389/fvets.2022.921720

**Published:** 2022-07-29

**Authors:** Kristina Tekavec, Tanja Švara, Tanja Knific, Jernej Mlakar, Mitja Gombač, Carlo Cantile

**Affiliations:** ^1^Department of Veterinary Science, University of Pisa, Pisa, Italy; ^2^Veterinary Faculty, Institute of Pathology, Wild Animals, Fish and Bees, University of Ljubljana, Ljubljana, Slovenia; ^3^Veterinary Faculty, Institute of Food Safety, Feed and Environment, University of Ljubljana, Ljubljana, Slovenia; ^4^Faculty of Medicine, Institute of Pathology, University of Ljubljana, Ljubljana, Slovenia

**Keywords:** dog, nerve sheath tumor, immunohistochemistry, methylation, H3K27me3

## Abstract

Nerve sheath tumors (NSTs) are characterized by neoplastic proliferation of Schwann cells, perineurial cells, endoneurial and/or epineurial fibroblasts. Diagnosis of NST is often challenging, particularly in distinguishing malignant NST (MNST) from other soft tissue sarcomas, or sometimes between low-grade MNST and benign NST. Recent studies in human pathology have demonstrated loss of trimethylation at lysine 27 of histone 3 (H3K27me3) in a subset of MNSTs using immunohistochemistry. Loss of H3K27me3 expression is rare in other high-grade sarcomas and also appears to be useful in distinguishing benign and low-grade MNSTs from high-grade subsets. In our retrospective study, we performed H3K27me3 immunohistochemistry in 68 canine tumors previously diagnosed as NST. We detected loss of H3K27me3 expression in 25% (*n* = 17) of all canine NST, including one neurofibroma, whereas 49% (*n* = 33) of tumors had mosaic loss of expression and 26% (*n* = 18) retained expression. No statistically significant differences were found between H3K27me3 expression, histopathological features of tumors, and their immunoreactivity for Sox10, claudin-1, GFAP, and Ki67. Because the classification of canine NST is not yet fully established and its correlation with the prognosis and clinical course of the disease is lacking, prospective studies with possible genetic analyses are needed to assess the true diagnostic value of H3K27me3 loss in canine NST.

## Introduction

Nerve sheath tumors (NSTs) are tumors that arise from Schwann cells, perineurial cells, and/or endo- or epineurial fibroblasts. Depending on the type of cells from which they originate, they are divided into four main subtypes: schwannoma, perineurioma and neurofibroma, which are benign, and malignant NST (MNST) (1). In dogs, NSTs are relatively rare tumors that arise most commonly in the spinal nerve roots, particularly in the cervicothoracic spinal segment, and brachial plexus (1, 2). In humans, they are often associated with genetic mutations and may also occur in a small percentage of cases after radiation therapy, whereas no such association has been demonstrated in dogs (3, 4). In cattle, a hereditary predisposition to NSTs similar to human neurofibromatosis is suspected, whereas in other animal species sporadic NSTs with unknown genetic background are occasionally described (5). The diagnosis of NSTs is often difficult, especially when it is necessary to distinguish MNSTs from other soft tissue sarcomas or, if it is necessary to discriminate the MNST that does not express obvious criteria of malignancy, from benign NSTs (4, 6). The location of the tumor contributes significantly to the diagnosis of NST; for instance, the involvement of a major nerve or the development of a malignant tumor in a previous benign NST are two indicators of MNST (6). In the absence of anatomic relationship with the nerve, the diagnosis may be made by considering morphological, immunohistochemical (IHC), and ultrastructural features of tumors, although these are often nonspecific and therefore of limited diagnostic value (1, 6, 7). A combination of IHC markers such as S100, Sox10, GFAP, claudin-1, laminin, and others, which may be selected depending on the differential diagnosis, can be used to confirm the nerve sheath origin of the tumor or to exclude other tumors (1, 4, 7).

Recent studies in human medicine have demonstrated loss of trimethylation at lysine 27 of histone 3 (H3K27me3) in a subset of MNSTs using IHC (8). H3K27me3 is a repressive histone modification associated with gene silencing (9). Responsible for H3K27me3 is a methyltransferase called Polycomb repressive complex 2 (PRC2), which is one of the two distinct multiprotein complexes with catalytic activity (10). Genetic alterations of embryonic ectoderm development (EED) and suppressor of zeste 12 (SUZ12), which are two of the core subunits of PRC2, result in loss of function of PRC2 and consequently loss of H3K27me3 (11). Because loss of H3K27me3 is rare in other high-grade sarcomas and, according to the studies, is also useful to distinguish benign and low-grade MNSTs from high-grade disease, in recent years it has been considered a valuable tool in the diagnosis of human NSTs, especially MNSTs, which usually have a worse prognosis (12–17). To our knowledge, only one study of H3K27me3 loss has been performed on a small number of MNSTs in dogs, and it revealed epigenetic subtypes similar to MNSTs in humans (18).

The aim of our study was to immunohistochemically evaluate the loss of H3K27me3 in a subset of canine NSTs using three different scoring scales. We have investigated the impact of previously determined tumor types and grades, including their histopathological and IHC features on H3K27me3 expression and evaluated the potential value of the latter for the diagnosis of NSTs in dogs.

## Materials and methods

### Tumor samples

The study was done on 68 canine tumors that had been previously diagnosed as NSTs based on their location, histopathological, and IHC features, assessed in the previous study by Tekavec et al. (19). The samples were collected between 2000 and 2022, and most of them came from the tissue archive of the Laboratory of Veterinary Neuropathology of the Department of Veterinary Science, University of Pisa (Italy), while one sample from 2021 came from the archive of the Institute of Pathology, Wild Animals, Fish and Bees of the Veterinary Faculty, University of Ljubljana (Slovenia).

All tumors were subjectively independently evaluated by veterinary pathologists involved in both studies (K.T., T.Š., M.G., C.C.). Inter-observer variation was minimal and discrepancies were re-evaluated for consensual definitive diagnosis. In a previous study, tissue and cellular characteristics of the tumors were evaluated, and IHC staining for Sox10, claudin-1, GFAP, CNPase, and the Ki-67 proliferation index was performed. Tissue characteristics included evaluation of tumor shape, demarcation, encapsulation, growth type, cellularity, growth pattern, amount and type of stroma, extent of necrosis, extent of hemorrhage, blood and lymphatic vessel invasion, herniation into vessels, inflammatory infiltrates, hyalinization, and osseous and cartilaginous components. Cellular characteristics included assessment of cell morphology, anisocytosis, anisokaryosis, cell margins, nuclear/cytoplasmic ratio, nuclear pleomorphism, nucleoli, number of mitoses per 10 high-power fields (HPF, 400× magnification −0.196 mm^2^), and presence of multinucleated cells (19). Details on the evaluation of the tissue and cellular criteria of tumors and evaluation of expression of IHC markers can be found in the Supplementary Tables 1, 2. In addition, MNSTs were classified into three histopathological grades according to the grading system originally used for cutaneous and subcutaneous soft tissue sarcomas (STS) in humans and dogs, which is based on tumor differentiation, number of mitoses, and intratumoral necrosis (6, 20, 21). Grading system for STS modified for MNST is provided in the Supplementary Table 3.

Although the study cited above included 79 NSTs, 11 tumors were subsequently excluded for this study due to lack of H3K27me3 expression in the internal positive controls.

### Immunohistochemistry

IHC for H3K27me3 was performed on 4 μm-thick formalin-fixed, paraffin-embedded tissue sections using the automated IHC stainer Ventana Benchmark XT (Ventana Medical Systems Inc., Tucson, Arizona, USA). After 64 min of antigen retrieval in CC1 antigen retrieval buffer (pH 8.5) at 25°C, the sections were incubated for 44 min at 37°C with a 1:400 diluted rabbit monoclonal antibody against H3K27me3 (clone C36B11; Cell Signaling Technology, USA). The Optiview DAB IHC Detection Kit (Ventana Medical Systems Inc., Tucson, Arizona, USA) was used to visualize the IHC reaction.

Human tonsil and canine lymph node were used as a positive controls and human diffuse midline glioma with a histone H3 K27M mutation and known H3K27me3 loss as a negative control (Supplementary Figure 1). The same tissues treated without primary antibody served as negative controls. Vascular endothelial cells and lymphocytes served as internal positive controls. In the absence of immunopositivity in the internal positive control, staining was repeated and samples that remained negative were excluded from the study.

### Evaluation of immunohistochemical expression

Immunohistochemical expression of H3K27me3 was independently evaluated by four veterinary pathologists (K.T., T.Š., M.G., C.C.) and one human pathologist (J. M.) without knowledge of clinicopathological data.

The nuclear expression of H3K27me3 was evaluated according to three different scoring scales previously described in human pathology:

1) The 4-tier scoring scale (22):
  - 3 +: Retained Expression in ≥ 95% of Tumor Cells (Diffuse Staining)
  - 2 +: Retained Expression in 50–94% of Tumor Cells (Loss in Minority)
  - 1 +: Retained Expression in 5–49% of Tumor Cells (Loss in Majority)
  - 0: retained expression in < 5% of tumor cells (complete loss).
2) The 3-tier scoring scale (12):
  - complete loss (0),
  - partial loss (1 + or 2 +) or
  - complete retention (3 +).
3) The 2-tier scoring scale (22):
  - complete loss (0) or
  - retention (> 0).

Partial loss, in which H3K27me3 expression was retained in 5–94% of tumor cells (1 + or 2 +), was also referred to as mosaic loss and was determined when immunopositive and immunonegative tumor cells were intermingled. When loss of H3K27me3 expression occurred in a well-defined area on a background of preserved expression, this was termed geographic loss, a variant of complete loss of expression, regardless of the percentage of tumor cells that retained expression (12).

Statistical analysis was performed separately for each of the three scoring scales, as described below.

### Statistical analysis

We assessed the difference in dog age, number of mitoses per 10 HPF and Ki-67 percentage between groups determined by tiers of H3K27me3 expression for all three scoring scales described above. We used the Wilcoxon rank sum test or the Kruskal-Wallis rank sum test, depending on the number of groups. Non-parametric tests were used because the variables were not normally distributed, which was tested with the Shapiro-Wilk normality test. We used Fisher's exact test to assess the association between H3K27me3 expression and other factor variables that included histopathological features (tissue and cellular characteristics of the tumors), grade of MNSTs, and results of immunohistochemical staining for Sox10, claudin-1 and GFAP. Because we made multiple comparisons within the data set, we applied a Benjamini-Hochberg correction to the p-values. A *p*-value < 0.05 was considered statistically significant. Statistical analysis was carried out with the statistical software R, version 4.1.2 (23).

## Results

### Clinicopathological data

Fifty male (74%) and 18 female (26%) dogs were included in this study, with a median age of 8 years (range of 1.5–13 years). Most of the dogs in the study were mixed breed (24/68, 35%). Besides, 23 different purebred dog breeds were represented, with Labrador Retriever (8/68) and German Shepherd (7/68) being the most common. Detailed information on other dog breeds is presented in Supplementary Table 2.

Most tumors were located at the roots of the spinal nerves (43/68, 63%). Ten tumors were located at the roots of the cervical spinal cord segment, 22 at the cervicothoracic, 4 at the thoracolumbar, and 6 at the lumbosacral spinal cord segment. In one dog, two lesions were noted, one in the thoracolumbar and one in the lumbosacral spinal cord segment. The second most common location was the brachial plexus (15/68, 22%), whereas a smaller number of NSTs involved the cranial nerves (3/68, 4%), appendicular nerves (5/68, 7%), and lumbar plexus (2/68, 3%).

Of 68 tumors, 59 (87%) were diagnosed as MNSTs. According to the grading system for STS, 14 (24%) of the MNSTs were histopathologically classified as low-grade (grade I), 23 (39%) as intermediate-grade (grade II), and 22 (37%) as high-grade (grade III). The majority of MNSTs (50/59, 85%) were conventional variants, whereas 4 had divergent differentiation, 4 had perineural differentiation, and 1 was epithelioid MNSTs. One high-grade conventional MNST was found to metastasize to the lung and brain. Of the 9 BNSTs (13%), 5 were neurofibromas, 3 were nerve sheath myxomas, and 1 was a schwannoma.

The details of the dogs included in the study, including available clinical data and final diagnoses, are provided in Supplementary Table 2. The histopathological and IHC features that led to the final diagnoses are described in detail in our previous study (19).

### Immunohistochemistry

The distribution of H3K27me3 staining scores are summarized in Table 1. Immunohistochemical staining results for H3K27me3 for each included case are provided in Supplementary Table 2.

**Table 1:** Immunohistochemical analysis of H3K27me3 expression in 68 canine nerve sheath tumors divided by type, subtype, and histological grade using the 4-tier scoring scale.

**Type**	**Subtype**	**Histological grade** ^*^	***N***	**Complete loss (** ***n*** **)**	**Loss in majority (** ***n*** **)**	**Loss in minority (** ***n*** **)**	**Complete retention (** ***n*** **)**
BNST	Neurofibroma	/	5	1	0	1	3
	Schwannoma	/	1	0	0	0	1
	Nerve sheath myxoma	/	3	0	0	2	1
	sum	/	9	1	0	3	5
MNST	Conventional	*Grade I*	14	2	1	6	5
		*Grade II*	20	5	2	9	4
		*Grade III*	16	5	5	3	3
		*sum*	50	12	8	18	12
	Divergent	*Grade III*	4	2	0	2	0
	Perineural	*Grade II*	2	1	1	0	0
		*Grade III*	2	1	1	0	0
		*sum*	4	2	2	0	0
	Epithelioid	*Grade II*	1	0	0	0	1
	sum	/	59	16	10	20	13
sum	/	/	68	17	10	23	18

*N, number of all samples of a certain type/subtype/histological grade; n, number of tumors with the indicated expression; H3K27me3, trimethylation at lysine 27 of histone 3; NST, nerve sheath tumor; BNST, benign nerve sheath tumor; MNST, malignant nerve sheath tumor*.

* *Histological grade only applies to MNSTs*.

**Figure 1:**
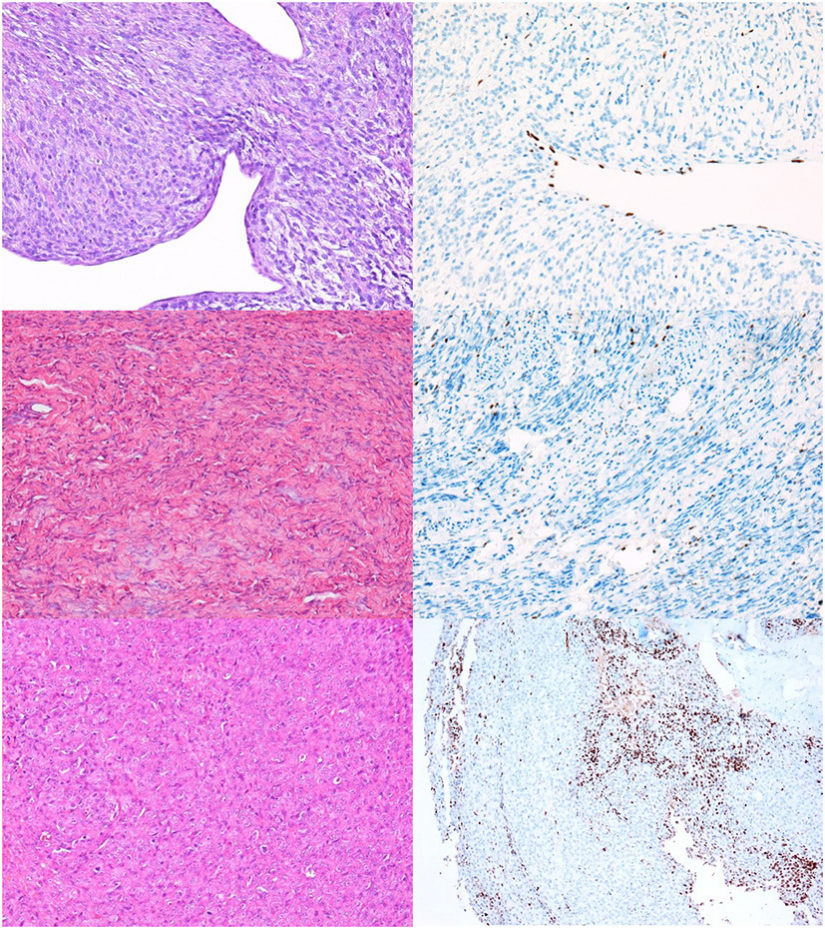
NSTs with complete loss of H3K27me3 expression. **(A,B)** MNST with perineural differentiation. HE, 200x **(A)**, and H3K27me3, 200x **(B)**. **(C,D)** Neurofibroma. HE, 200x **(C)**, and H3K27me3, 200x **(D)**. In Figures **(B,D)** only endothelial cells and scattered lymphocytes express H3K27me3, whereas tumor cells are immunonegative. **(E,F)** Conventional MNST, HE, 200x **(E)**. Tumor exhibits geographical loss of H3K27me3 expression, H3K27me3, 100x **(F)**.

Complete loss of H3K27me3 expression was detected in 25% (17/68) of all NSTs in our study (Figure 1), including one neurofibroma (Figures 1C,D). In two cases, we detected a geographic loss of staining (Figures 1E,F), which was considered a complete loss of expression.

**Figure 2:**
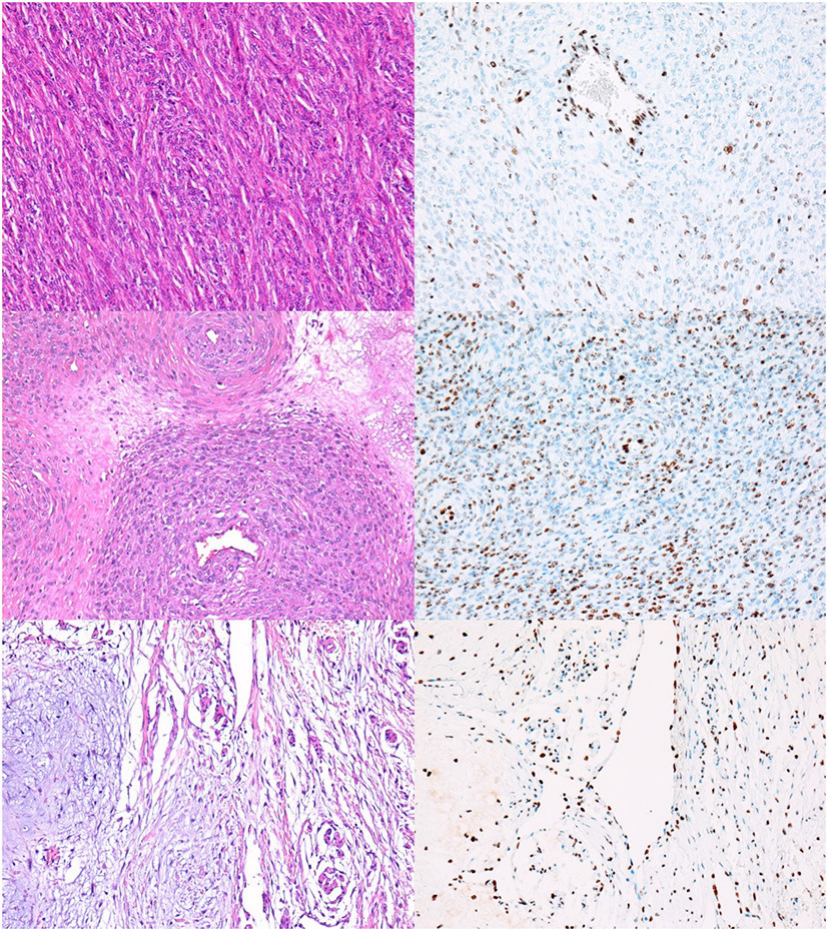
NSTs with mosaic loss of H3K27me3 expression. **(A,B)** MNST with perineural differentiation. HE, 200x **(A)**, with loss of H3K27me3 expression in 50–94% of tumor cells, H3K27me3, 200x **(B)**. Conventional MNST, HE, 200x **(C)**, with loss of H3K27me3 expression in 5–49% of tumor cells, H3K27me3, 200x **(D)**. Nerve sheath myxoma, HE, 200x **(E)**, with loss of H3K27me3 expression in 5–49% of tumor cells, H3K27me3, 200x **(F)**.

We found a high rate of mosaic loss of expression, which was observed in 48% of all NSTs (33/68) (Figure 2). Mosaic loss was observed in 51% of malignant (Figures 2A–D) and 33% of benign NSTs (Figures 2E,F).

**Figure 3:**
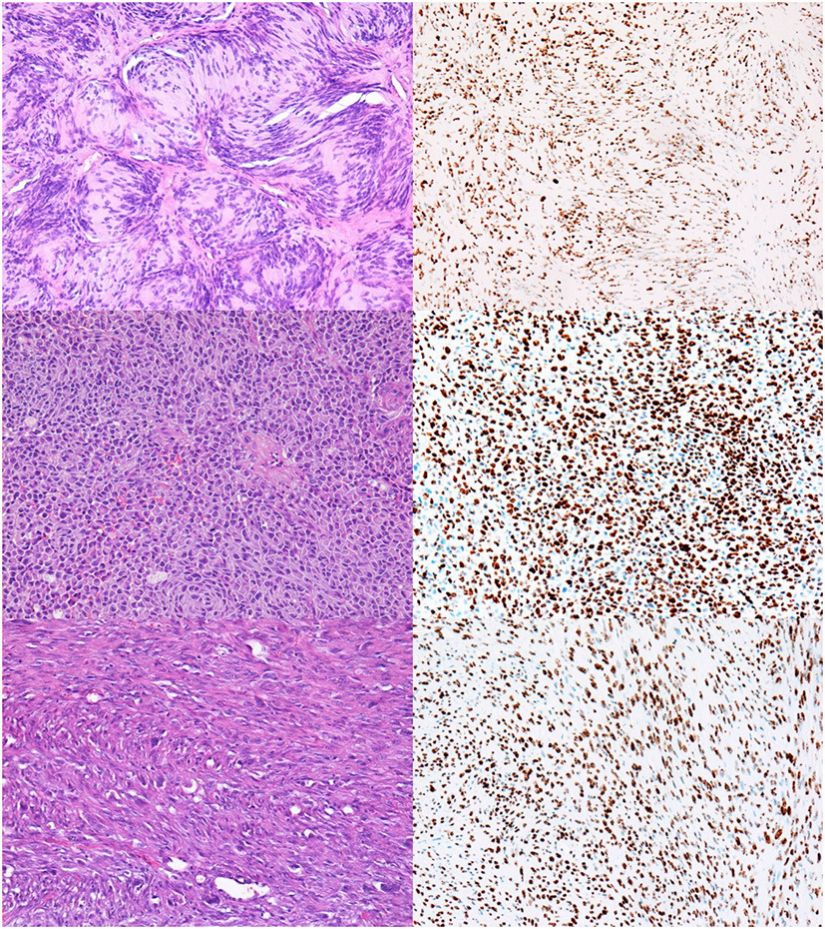
NSTs with complete retention of H3K27me3 expression. **(A,B)** Classic schwannoma with Verocay bodies. HE, 200x **(A)** and H3K27me3 **(B)**. **(C,D)** Epithelioid MNST. HE, 200x **(C)** and H3K27me3, 200x **(D)**. **(E,F)** Conventional MNST with confirmed metastases. HE, 200x **(E)** and H3K27me3 **(F)**.

Twenty-six percent of tumors showed complete retention of staining in ≥ 95% of tumor cells (Figure 3); 56% of BNSTs and 22% of MNSTs, including epithelioid MNST (Figures 3C,D) and MNST with confirmed metastases (Figures 3E,F).

No significant association between age, sex or breed of included dogs, or tumor location and the tier of H3K27me3 expression was found for none of the three scoring scales. There was also no association between H3K27me3 status and the type or grade of MNSTs included in the study. In addition, there were no statistically significant association between H3K27me3 expression and histopathological and IHC features of the tumors.

Of seven samples included in the study that were negative for Sox10, claudin-1, and GFAP on IHC staining, one had complete loss of H3K27me3 expression. Four of these cases had partial loss and two had complete retention of H3K27me3 expression.

**Figure 4:**
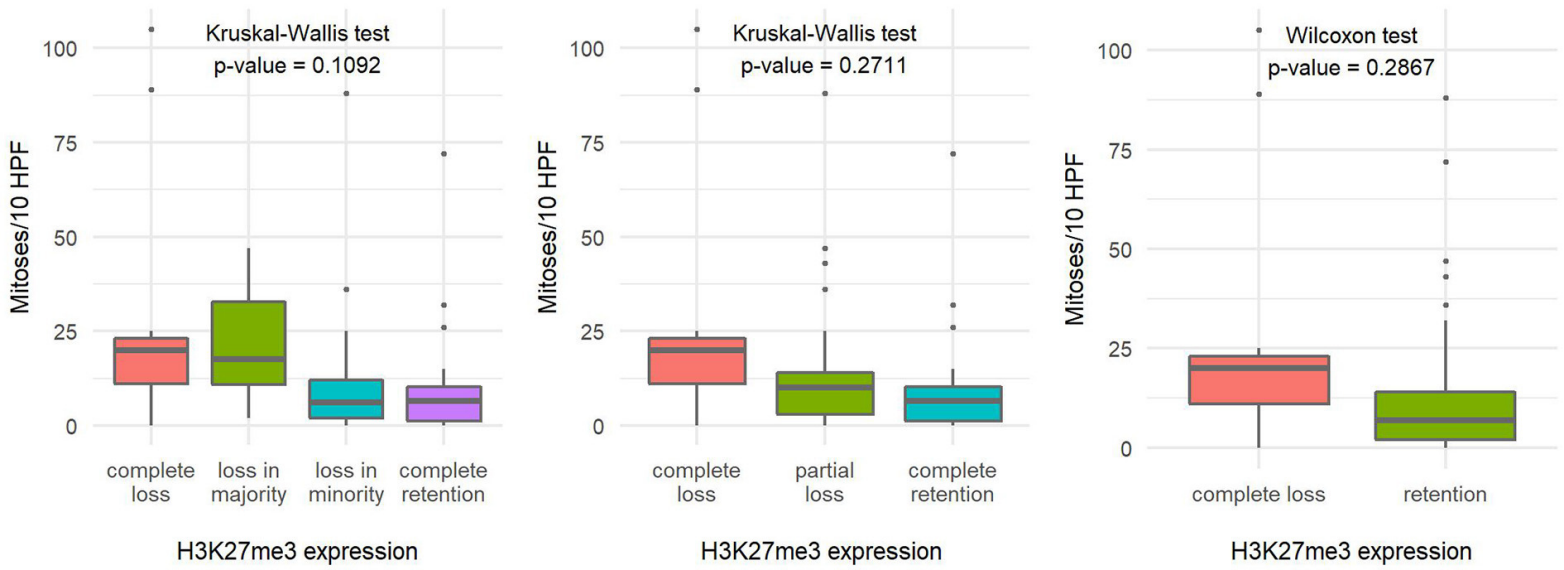
Comparison of the number of mitoses per ten high-power fields based on H3K27me3 expression using a 4-tier scoring scale **(A)**, a 3-tier scoring scale **(B)**, and a 2-tier scoring scale **(C)**, indicating the statistical test used and the corresponding *p*-value.

**Figure 5:**
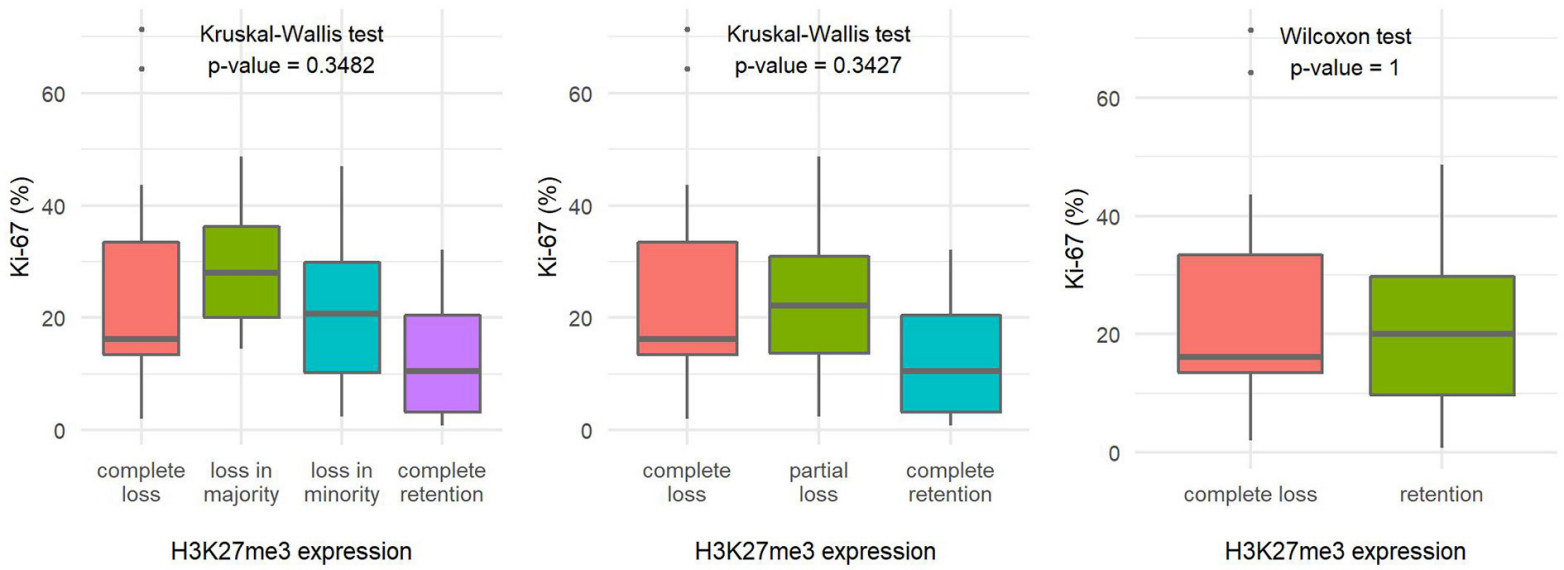
Comparison of the Ki-67 proliferation index fields based on H3K27me3 expression using a 4-tier scoring scale **(A)**, a 3-tier scoring scale **(B)**, and a 2-tier scoring scale **(C)**, indicating the statistical test used and the corresponding *p*-value.

The number of mitoses per 10 HPF and the Ki-67 proliferation index in tumors with loss of H3K27me3 expression appeared to be slightly higher compared with tumors with retained H3K27me3 expression, but the difference in both variables proved to be statistically insignificant (Figures 4, 5).

## Discussion

Diagnosis of NSTs remains challenging for pathologists because their morphologic and IHC features are often not specific. While the classification of NSTs in human pathology is more frequently updated, and takes into account the prognostic data (3, 24), such data are still lacking in veterinary pathology. Recent studies in human pathology have shown that loss of H3K27me3 is useful to distinguish MNSTs from their histological mimics and, according to some studies, can also serve as a negative prognostic indicator for human NSTs (8, 16), although the results on sensitivity and specificity of this marker vary between studies (22). In this study, we immunohistochemically investigated the expression of H3K27me3 in 68 canine NSTs that had been diagnosed, classified, and, if malignant, graded based on their histopathologic and IHC features in our previous study (19). We compared the individual histopathological features and IHC staining results for Sox10, claudin-1, GFAP, and proliferation index Ki-67 with H3K27me3 expression to assess the potential diagnostic value of the latter.

We detected loss of H3K27me3 expression in 25% of NSTs included in the study (17/68), with 16 tumors being malignant and one classified as benign—neurofibroma. In two MNSTs, we detected a geographic loss of staining, which we considered a variant of complete loss, following the interpretation of Asano et al., who assumed that complete loss of H3K27me3 expression in well-defined areas on a background of preserved expression indicates PRC2 inactivation (12). When comparing histopathological and IHC features of MNSTs with H3K27me3 expression, we found no significant association between H3K27me3 loss and any variable. Although we found a slightly higher Ki-67 proliferation index and a higher number of mitoses per 10 HPF in tumors with loss of H3K27me3 expression compared with tumors with partial or complete retention of the marker, the results were not statistically significant. Unfortunately, we lacked data on the proliferation marker Ki-67 for 17 of the included tumors, 16 of which were malignant and one benign. According to our previous study, staining of these samples was assessed as unreliable, probably due to poor antigen preservation in old archive blocks (19, 25).

Previous studies from human pathology have found loss of H3K27me3 expression in a higher percentage of MNSTs, but results vary depending on the study. Cleven et al. reported loss of H3K27me3 expression in 34% (55/162) of MNSTs (13), while Le Guellec et al. found 72% (88/122) of MNSTs with loss of expression in their study (14). The results of other studies fall somewhere in between (8, 12, 16, 26). Lu et al. performed meta-analysis of available data from the literature and estimated the incidence of H3K27me3 loss in MNSTs to be 53% (27). In humans, loss of H3K27me3 expression is common in MNSTs induced by radiotherapy, with loss of expression occurring in 86-100% of cases (27). While some authors reported a higher incidence of H3K27me3 loss in NF1-associated tumors (8, 26, 28), others found it more common in sporadic MNSTs (12, 16). Based on the processed data, Lu et al. estimated H3K27me3 loss as a feature of ~50% of sporadic and NF-1-associated tumors (27). In veterinary medicine, data on genetic disorders associated with neoplasm occurrence are generally lacking, and no correlation can be made between H3K27me3 expression and the genetic profile of dogs with NSTs.

To our knowledge, no study from human pathology has reported a complete loss of H3K27me3 expression in neurofibroma. They even describe that this marker has the potential to help differentiate between MNSTs and BNSTs (12, 13, 26). Cleven et al. were the only authors to report complete loss of expression in a case of schwannoma (1/44) (13). Such results raise questions about the diagnosis of neurofibroma with loss of H3K27me3 expression in our study. The diagnosis was previously made based on the histopathological and IHC features of the tumor, all of which were suggestive of its benign nature (19), but given the lack of data on the further clinical course of the disease and survival of the dog, we cannot completely rule out the possibility of low-grade MNST. However, until a larger prospective study of canine NSTs is conducted, we also cannot be certain that the interpretation of the results of NSTs in dogs can fully follow those in human medicine.

In our study, we did not evaluate the specificity of H3K27me3 loss because no other type of tumor was included. Most studies from human pathology show specificity above 90%, with the exception of the study of Le Guellec et al., who detected the H3K27me3 loss also in 25% (7/29) of melanomas and states specificity of 75% (14, 27). Other authors also report individual cases of other non-MNSTs with the complete loss of H3K27me3 expression, such as undifferentiated pleomorphic sarcoma, radiation-induced osteosarcoma (12), dedifferentiated chondrosarcoma (29), synovial sarcoma, fibrosarcomatous dermatofibrosarcoma protuberans, angiosarcoma, and clear cell sarcoma (13).

Some studies from human pathology showed complete loss of H3K27me3 in 89–100% of MNSTs with divergent differentiation, suggesting its potential utility for distinguishing such tumors from osteosarcomas and rhabdomyosarcomas when location and morphology pose a challenge for differentiation (12, 16, 26). Nevertheless, caution is warranted; Makise et al. found loss of H3K27me3 expression in 6% (3/47) of cases of dedifferentiated chondrosarcomas, all of which had heterologous differentiation (29). Since PRC2 and H3K27me3 play an important role in maintaining and reprogramming cell identity during normal development, epigenetic alteration caused by deficiency of H3K27me3 could lead to heterologous differentiation, regardless of tumor type (29). Cleven et al. found retention of H3K27me3 expression in all MNSTs with divergent differentiation (13). In the study by Ersen et al., the loss was also not a feature of MNST with rhabdomyoblastic differentiation, and in MNSTs with glandular differentiation, expression was retained in the malignant glandular component (8). Of the four MNSTs with divergent differentiation in our study, two showed complete loss of H3K27me3 expression, while two showed only partial loss of expression in <50% of tumor cells.

Of the seven cases in which expression of Sox10, claudin-1, CNPase, and GFAP was negative (19), one also had loss of H3K27me3 expression. However, to date, there is insufficient evidence to take this finding as confirmation of MNST in dogs.

In our case series, there was only one epithelioid MNST that showed a typical histopathological and IHC phenotype corresponding to the human counterpart. Consistent with the results of previous human pathology studies, it resulted in complete retention of H3K27me3 expression (12, 16, 26, 28). This result proves once again that epithelioid MNSTs have distinct morphological, IHC, and molecular characteristics compared to non-epithelioid MNSTs (16).

Including epithelioid MNST, only 26% of NSTs (18/68) in our study had intact expression, 56% of BNSTs and 22% of MNSTs. A large number of tumors in our study (49%) showed mosaic loss of H3K27me3 expression, including 51% of MNST and 33% of BNST. Although it was initially assumed that the mosaic loss of H3K27me3 expression was specific to MNSTs, subsequent studies have shown otherwise. The mosaic loss of staining has been described in neurofibromas, and such a staining pattern has also been demonstrated in a number of other tumor types (12, 26). The authors note that such a result may not be related to a PRC2 mutation and emphasize that this should not be considered diagnostic for MNSTs. Only a complete loss of staining, which may be global or geographic, should be considered significant for the usefulness of H3K27me3 loss in the diagnosis of MNST (12). Furthermore, it should be used in the context of a broad IHC panel to aid in the diagnosis of MNST (9).

The loss of H3K27me3 was associated with a worse prognosis in human patients with breast, ovarian, and pancreatic cancers (30). The study by Cleven et al. associated the loss of H3K27me3 with shorter survival time (13), while the study by Pekmezci et al. found no significant association between loss of H3K27me3 and clinical outcome in MNSTs (22). Most other studies in human medicine have not performed survival analysis based on H3K27me3 loss (27). Because our study is retrospective and we unfortunately lack data on disease progression and survival of the dogs, we cannot relate H3K27me3 expression to tumor prognosis. Although in our previous study we suggested that some histopathological and IHC features of tumors could help predict their biological behavior and proposed to update the classification based on the latest human WHO classification, prospective studies are needed to find the most reliable prognostic indicators (19). We believe that IHC for H3K27me3 may add some value to the diagnosis of NST in dogs and that this marker may prove useful if included in the prospective study.

In conclusion, we demonstrated a complete loss of H3K27me3 expression in a subset of canine NSTs. Our results showed no significant association between H3K27me3 expression and histopathological or IHC features of the included tumors. For a reliable evaluation of the usefulness of H3K27me3 loss in the diagnosis of NSTs, a prospective study investigating the association of H3K27me3 expression with the clinical course of the disease and survival time is needed in combination with genetic testing. In addition, H3K27me3 expression should also be studied in a spectrum of other canine tumor types, especially in the most common differential diagnoses of NSTs, to test the specificity of this marker.

## Data availability statement

The original contributions presented in the study are included in the article/Supplementary material, further inquiries can be directed to the corresponding author/s.

## Ethics statement

Ethical review and approval was not required for the animal study because the samples included in this study were submitted to the laboratory for routine diagnostic procedures. Written informed consent was obtained from the owners for the participation of their animals in this study.

## Author contributions

KT, TŠ, JM, MG, and CC: conceptualization, review, and editing. KT and CC: data collection, and curation. TK: statistical analysis. KT and TK: visualization. KT: original draft preparation. TŠ, MG, and CC: supervision. All authors contributed to the article and approved the submitted version.

## Funding

Ph.D. grant from University of Pisa. The research was also partially funded by the Slovenian Research Agency, Grant Number P4-0092.

## Conflict of interest

The authors declare that the research was conducted in the absence of any commercial or financial relationships that could be construed as a potential conflict of interest.

## Publisher's note

All claims expressed in this article are solely those of the authors and do not necessarily represent those of their affiliated organizations, or those of the publisher, the editors and the reviewers. Any product that may be evaluated in this article, or claim that may be made by its manufacturer, is not guaranteed or endorsed by the publisher.
