# Supplementary material for: Loss of H3K27me3 expression in canine nerve sheath tumors
Source: Front Vet Sci. 2022 Jul 29;9:921720. doi: 10.3389/fvets.2022.921720 (PMC9372589; doi:10.3389/fvets.2022.921720)
Supplement: Supplementary file 1 [file Data_Sheet_1.docx]

**Supplementary Table 1**: Evaluation of the tissue and cellular criteria of nerve sheath tumors, summarized after the previous study of Tekavec et al. (19).

| **TISSUE CRITERIA** | | |
| --- | --- | --- |
| Circumscription | - Well circumscribed - Ill-defined | |
| Encapsulation | - Encapsulated - Capsule invaded - Incomplete fibrous pseudocapsule - Unencapsulated | |
| Shape | - Nodular / multilobular / cystic / other: | |
| Cellularity | - Low - Moderate - High | |
| Growth pattern;  The proportion of each growth pattern  ((0) absent, (1) <25 %, (2) 25 – 50 %, (3) 50 – 75 %, (4) > 75 %) | Overall microscopical appearance:   - Heterogeneous - Uniform - Antoni type A (interlacing bundles, storiform, concentric) - Antoni type B (loose textured)   Sheets / cords / meshwork of reticular fibers / rossettelike formations / sweeping fascicles / whorls / storiform pattern /plexiform pattern / other: | |
| Stroma | Amount:   - Small - Moderate - Large   Type of stroma: collagenous / myxoid / fine /coarse / fibrovascular / fibrous / other: | |
| Necrosis | - Absent - Present, ≤ 50 % - Present, > 50 % | |
| Hemorrhages | - Absent - Present | |
| Vascular invasion | - Absent - Blood vessel invasion - Lymphatic vessel invasion - Blood and lymphatic vessel invasion | |
| Herniation into vessels | - Absent - Present | |
| Inflammatory infiltrates | - Absent - Present - Type: ________________________ - The extent of inflammation - Location (perivascular, within the capsule, …): | |
| Hyalinization | - Absent - Present | |
| Osteoid components | - Absent - Present | |
| Cartilaginous components | - Absent - Present | |
| **CELLULAR CRITERIA** | | |
| Cellular morphology | Spindle / oval / polygonal / fusiform / onion-bulb-like formations / signet ring-like / epithelioid / other: | |
| Anisocytosis | - Mild - Moderate - Strong | |
| Anisokaryosis | - Mild - Moderate - Strong | |
| Nuclear characteristics | Nuclear pleomorphism:   - Absent - Present: - Mild - Moderate - Strong   Nuclear shape: oval / round / elongated / bean-shaped / vesicular / hyperchromatic / other: | |
| Nucleoli | - Not evident - Prominent - Number of nucleoli – up to ­­­_______ - Shape of nucleoli | |
| Cytoplasm | Amount:   - Small - Moderate - Large | Color:  Character:   - *Homogenous* - *Granular* - *Containing pigment* - *Other:* |
| Cell borders | - Distinct - Indistinct | |
| Mitoses  ((1) 0-9/10 HPF, (2) 10-19/10 HPF, (3) >19/10 HPF) | 1/ 2/ 3/ 4/ 5/  6/ 7/ 8/ 9/ 10/  MI = ______/10 HPF  Max. number of mitoses per HPF:  Presence of atypical mitotic figures: | |
| Multinucleated cells | - Absent - Present 🡪 Up to ________ nuclei | |

**Supplementary Table 2**: Signalment, clinical features, and tumor location of dogs included in the study, with final diagnosis including immunohistochemical staining for Sox10, claudin-1, GFAP, Ki-67 (19), and H3K27me3.

| No. | Breed | Age (years) | Sex | Clinical presentation | Location | Tumor type | Histological grade (if malignant) | Sox10  expression | Claudin-1  expression | GFAP  expression | Proliferation index Ki-67 (%) | H3K27me3 expression |
| --- | --- | --- | --- | --- | --- | --- | --- | --- | --- | --- | --- | --- |
| 1 | Mixed breed | 8 | M | Lameness/paresis of the thoracic limbs (LMN and UMN type). | Below the spine at the level of C6-T1, extending upward through the foramina and infiltrating the epidural space. | MNST – conventional | III | − | − | − | ND | Mosaic loss – loss in minority |
| 2 | Mixed breed | 7.5 | F | Atrophy of the right shoulder.Right pleurothotonus | Extra- and intradural lesion at the level of C6-C7 | MNST – conventional | II | ++ | ++ | + | ND | Complete loss |
| 3 | Mixed breed | 8 | M | Paraplegia (LMN type) and absence of deep pain perception. | Lumbosacral extra- and intradural lesion | MNST – conventional | II | + | + | − | ND | Mosaic loss – loss in majority |
| 4 | English Setter | 7 | M | Postural deficit, hyporeflexia of the right forelimb. | Nerve roots involvement at the level of the cervicothoracic spine. | MNST – conventional | III | (++) | (++) | (++) | ND | Complete loss |
| 5 | Beagle | 8 | M | NR | Brachial plexus. | MNST – perineural | II | − | +++ | − | ND | Complete loss |
| 6 | Mixed breed | 8 | M | NR | Nerve roots at the level of C7-T1 | MNST – conventional | I | ++ | ++ | ++ | ND | Complete retention |
| 7 | German Wirehaired Pointer | 8 | M | Left forelimb paresis.  Absence of spinal reflexes.  Pain on palpation of the axilla. | Extramedullary centripetal lesion at the root of the left radial nerve. | MNST – conventional | II | + | + | + | ND | Complete loss |
| 8 | Shih-Tzu | 4 | F | Pulmonary and brain metastases. | Brachial plexus. | MNST – conventional | III | − | − | − | ND | Complete retention |
| 9 | Newfoundland dog | 1,5 | M | Lameness of the left hindlimb with impaired proprioception. | Tumor located ventral to the left transverse process of L7, adjacent to the nerve root L6. The tumor is encapsulated proximally and continues distally within the nerve. | Nerve sheath myxoma | NA | + | + | + | ND | Mosaic loss – loss in minority |
| 10 | Yorkshire Terrier | 7 | M | NR | Intradural extramedullary lesion at the level of C7-T1. | MNST – conventional | I | ++ | ++ | (+) | ND | Mosaic loss – loss in minority |
| 11 | Labrador Retriever | 10 | M | NR | Extradural mass at the level of L1-L2 – lateralized on the left. | MNST – conventional | II | − | + | − | ND | Complete retention |
| 12 | Mixed breed | 11 | M | Chronic lameness and paresis of the left forelimb. EMG: denervation atrophy. | Nerve roots – T1. | MNST – conventional | I | − | − | − | ND | Mosaic loss – loss in minority |
| 13 | Labrador Retriever | 7 | F | Lameness and muscle atrophy of the right pelvic limb | Right femoral nerve. | MNST – conventional | I | ++ | − | − | ND | Complete loss |
| 14 | Labrador Retriever | 6 | M | Head tilt to the left. | Left V. nerve. | MNST – conventional | III | + | + | − | ND | Mosaic loss – loss in majority |
| 15 | Mixed breed | 8 | M | Neck pain and lameness of the right forelimb. | Intradural extramedullary neoplasia of the roots C6-C7. | MNST – conventional | II | +++ | + | − | ND | Complete retention |
| 16 | German Shepherd | 12 | M | NR | Nerve root C8. | MNST – conventional | I | + | +++ | + | ND | Complete retention |
| 17 | German Shepherd | 12 | M | Progressive left hemiparesis, progressing to recumbency. | Extra- and intravertebral neoplasm at the level of the left foramina C5-C6. | MNST – conventional | III | + | + | − | ND | Complete loss |
| 18 | French Bulldog | 6 | M | NR | Lateral mass on the left *medulla oblongata* and *pons*. | MNST – conventional | III | + | + | + | 11.8 | Complete retention |
| 19 | Labrador Retriever | 6 | M | NR | Right brachial plexus. | MNST – conventional | I | ++ | ++ | + | 9.1 | Complete retention |
| 20 | Maltese | 6 | F | NR | Involvement of the nerve roots C7-T1. | MNST – conventional | II | ++ | ++ | + | 23.2 | Complete loss |
| 21 | Yorkshire Terrier | 12 | F | NR | Intramedullary lesions at the level of C2 and C6. | MNST – conventional | II | + | − | − | 15.8 | Complete loss |
| 22 | Labrador Retriever | 5 | M | NR | Left sciatic nerve. | MNST – conventional | I | + | + | + | 14.4 | Mosaic loss – loss in majority |
| 23 | Mixed breed | 6 | F | Atrophy of the muscles of the shoulder and left forelimb. | Tumor of the nerve roots at the cervicothoracic spinal cord. | MNST – conventional | I | ++ | ++ | ++ | 10.5 | Complete retention |
| 24 | Mixed breed | 6 | M | NR | Left brachial plexus. | MNST – conventional | III | + | + | − | 28.5 | Mosaic loss – loss in majority |
| 25 | English Setter | 11 | M | NR | Neoplasia of the left brachial plexus (C7-T1). | MNST – perineural | III | − | +++ | − | 20.1 | Mosaic loss – loss in majority |
| 26 | Czechoslovakian Wolfdog | 11 | M | NR | Nerve root C8. | MNST – perineural | II | − | +++ | − | 35.3 | Mosaic loss – loss in majority |
| 27 | Boston Terrier | 8 | M | NR | Brachial plexus. | MNST – conventional | III | ++ | + | − | 21.0 | Complete loss |
| 28 | Labrador Retriever | 4 | M | NR | Lardaceous extradural neoplasia C1-C2. | MNST – conventional | III | − | − | − | 27.7 | Complete retention |
| 29 | Mixed breed | 9 | M | NR | Brachial plexus. | MNST – perineural | III | − | +++ | − | 64.3 | Complete loss |
| 30 | Rottweiler | 9 | F | NR | Tumor of the right C1-C2. | Schwannoma – classic | NA | +++ | − | ++ | 1.5 | Complete retention |
| 31 | German Shepherd | 12 | M | NR | Left radial nerve. | MNST – conventional | I | + | − | − | 15.7 | Complete loss |
| 32 | German Shepherd | 4 | M | NR | Medullary lesion of the cervical spine. | MNST – conventional | II | + | + | − | 9.1 | Mosaic loss – loss in minority |
| 33 | Mixed breed | 11 | M | NR | Nerve root of the left C7. | MNST – conventional | I | ++ | ++ | + | 14.2 | Mosaic loss – loss in minority |
| 34 | West Highland White Terrier | 10 | M | NR | Lumbosacral plexus. | MNST – conventional | II | − | − | − | 27.4 | Mosaic loss – loss in majority |
| 35 | German Shepherd | 8 | M | NR | Brachial plexus. | MNST – divergent | III | − | ++ | − | 22.1 | Mosaic loss – loss in minority |
| 36 | Beagle | 8 | M | NR | Intradural extramedullary neoplasia C4-C5. | Nerve sheath myxoma | NA | ++ | ++ | ++ | 3.2 | Complete retention |
| 37 | Mixed breed | 7 | M | NR | Tumor of the nerve roots at the cervicothoracic spinal cord. | MNST – conventional | II | ++ | ++ | + | 45.3 | Mosaic loss – loss in minority |
| 38 | Mixed breed | 13 | F | NR | T13 and L5 nerve roots. | MNST – conventional | II | − | ++ | − | 7.8 | Mosaic loss – loss in minority |
| 39 | Mixed breed | 11 | M | NR | Right brachial plexus. | MNST – conventional | I | + | ++ | − | 29.7 | Mosaic loss – loss in minority |
| 40 | Labrador Retriever | 7 | M | NR | Endocanalar, extramedullary C6 lesion. | MNST – conventional | II | + | + | + | 23.5 | Mosaic loss – loss in minority |
| 41 | West Highland White Terrier | 12 | M | Progressive hemiparesis for 15 days. | Intradural extramedullary mass involving the nerve roots at the level of the cervicothoracic spinal cord. | MNST – conventional | I | ++ | ++ | − | 16.4 | Mosaic loss – loss in minority |
| 42 | Mixed breed | 11 | M | Progressive lameness of left forelimb. | A mass in the left shoulder region – involving the brachial plexus and cervicothoracic spinal cord C4-T7. | MNST – conventional | II | ++ | ++ | − | 30.2 | Complete retention |
| 43 | Cane Corso | 8 | M | Bilateral flexor hyporeflexia and proprioceptive deficit of the right forelimb. Neck pain. | Neoplasia of the right C4 with medullary infiltration. | MNST – conventional | III | − | + | − | 39.2 | Mosaic loss – loss in majority |
| 44 | Mixed breed | 12 | F | NR | Left trigeminal nerve. | MNST – conventional | II | + | ++ | − | 11.1 | Complete loss |
| 45 | Mixed breed | 9 | M | NR | Nerve roots involvement at the level of the lumbosacral spinal cord. | MNST – conventional | III | − | + | − | 16.2 | Complete loss |
| 46 | Mixed breed | 11 | M | NR | Left brachial plexus. | MNST – conventional | III | + | ++ | − | 46.4 | Mosaic loss – loss in minority |
| 47 | Mixed breed | 4 | M | Paraparesis with proprioceptive deficit, urinary and fecal incontinence. | Intradural, intramedullary mass L4-L7. | MNST – conventional | I | + | (+) | (+) | 2.4 | Mosaic loss – loss in minority |
| 48 | Dogo Argentino | 5 | F | NR | Tumor of the right C6. | Nerve sheath myxoma | NA | ++ | ++ | + | 8.2 | Mosaic loss – loss in minority |
| 49 | Mixed breed | 11 | F | Right forelimb lameness, decreased proprioception and pain. Right Horner syndrome. Absence of panniculus reflex cranial to right T11. | Right axillary mass extending to the spinal cord by multiple nerve roots. | MNST – divergent | III | ++ | ++ | + | 71.4 | Complete loss |
| 50 | Bernese Mountain dog | 12 | M | NR | Brachial nerve. | MNST – divergent | III | ++ | − | − | 11.2 | Complete loss |
| 51 | Labrador Retriever | 3 | M | NR | Lesion of the T9-T10. | Neurofibroma | NA | − | ++ | + | 0.8 | Complete retention |
| 52 | German Shepherd | 10 | F | Right forelimb lameness, flexor areflexia, and muscular atrophy. | Right C8 nerve. | Neurofibroma – plexiform | NA | ++ | ++ | ++ | 0.9 | Complete retention |
| 53 | Boxer | 6 | M | Chronic lameness and pain of the left forelimb. | Left ulnar nerve. | MNST – epithelioid | II | +++ | − | − | 20.4 | Complete retention |
| 54 | French Bulldog | 8 | F | NR | Neoplasia of C2 with compression of the spinal cord. | MNST – conventional | I | ++ | ++ | − | 20.1 | Complete retention |
| 55 | German Shepherd | 7 | F | Left forelimb paresis and hyporeflexia. | Left C8 nerve root. | MNST – conventional | III | ++ | +++ | − | 48.7 | Mosaic loss – loss in majority |
| 56 | Papillon | 7 | M | Progressive pain of the right forelimb (radicular syndrome) and neck pain. | Right brachial plexus tumor (C1-T2). | MNST – divergent | III | ++ | ++ | ++ | 31.7 | Mosaic loss – loss in minority |
| 57 | Mixed breed | 11 | M | Right paraparesis, ataxia, and proprioceptive deficit. | Nerve root T13. | MNST – conventional | II | + | − | − | 11.3 | Mosaic loss – loss in minority |
| 58 | Bernese Mountain dog | 7 | M | Ataxia of the four limbs and neck pain. | Cervicothoracic spinal cord segment | Neurofibroma | NA | ++ | + | ++ | 2.0 | Complete loss |
| 59 | Mixed breed | 9 | M | NR | Lumbar plexus (L6-L7). | MNST – conventional | II | ++ | + | + | 30.1 | Mosaic loss – loss in minority |
| 60 | Mixed breed | 10 | M | NR | Neoplasia of the left root L3. Invasion of the spinal canal – intramedullary growth. | MNST – conventional | II | + | ++ | + | 20.7 | Mosaic loss – loss in minority |
| 61 | Mixed breed | 8 | F | Chronic paresis of the left forelimb. | Left brachial plexus. | MNST – conventional | II | + | + | + | 47.0 | Mosaic loss – loss in minority |
| 62 | Cavalier King Charles Spaniel | 12 | M | NR | Lumbar paravertebral lesion on the left side. | MNST – conventional | III | + | + | + | 13.0 | Mosaic loss – loss in minority |
| 63 | Golden Retriever | 4 | M | Progressive tetraparesis. | Epidural lesion C2-C4. | MNST – divergent | III | − | − | − | 43.6 | Complete loss |
| 64 | Staffordshire Terrier | 8 | F | Right hindlimb paresis. | Extramedullary mass at the level of the L4-L5 nerve roots. | MNST – conventional | III | − | − | − | 19.6 | Mosaic loss – loss in majority |
| 65 | Staffordshire Terrier | 9 | M | NR | Neoplasia of the right C2 root with endocanalar extension and spinal cord compression. | Neurofibroma | NA | ++ | +++ | ++ | 10.0 | Complete retention |
| 66 | French Bulldog | 10,5 | F | NR | Neoplasia of the right C7 root. | MNST – conventional | II | + | + | − | 32.1 | Complete retention |
| 67 | Mixed breed | 6 | F | Progressive ataxia with severe proprioceptive deficits and cervical pain. | Intradural, extramedullary mass at the level of right C2. | Neurofibroma | NA | ++ | ++ | + | 6.4 | Mosaic loss – loss in minority |
| 68 | Jack Russel Terrier | 6,5 | M | Chronic lameness of the right forelimb. | Neoplasia of the right brachial plexus extending to the C6-T1 nerve roots. | MNST - conventional | II | + | +++ | ++ | 29.3 | Mosaic loss – loss in minority |

M: male; F: female; LMN: lower motor neuron; UMN: upper motor neuron; EMG: electromyography; NR: not reported; MNST: malignant nerve sheath tumor; BNST: benign nerve sheath tumor; -: negative reaction; +: weak positive reaction; ++: moderate positive reaction; +++: strong positive reaction; ND: no data; NA: not applicable.

The result in brackets indicates that the reaction may be limited to the nerve residues.

**Supplementary Table 3**: Grading system for STS modified for MNST by Rodriguez et al. (6, 19).

| *Differentiation score* | |
| --- | --- |
| 1 | Well differentiated MNST arising in transition from neurofibroma |
| 2 | Conventional, monomorphous spindle cell MNST |
| 3 | Highly pleomorphic MNSTs, as well as MNST with divergent differentiation |
| *Mitotic count* | |
| 1 | 0–9 mitoses/10 HPF |
| 2 | 10–19 mitoses/10 HPF |
| 3 | >19 mitoses/10 HPF |
| *Tumor necrosis* | |
| 0 | No necrosis |
| 1 | ≤ 50% necrosis |
| 2 | > 50% necrosis |
| *HISTOLOGICAL GRADE** | |
| I | ≤ 3 |
| II | 4–5 |
| III | ≥ 6 |

STS: soft tissue sarcoma. MNST: malignant nerve sheath tumor. HPF: high-power fields.

*Histological grade corresponds to the sum of all three parameters assessed – differentiation score, mitotic count, and tumor necrosis.


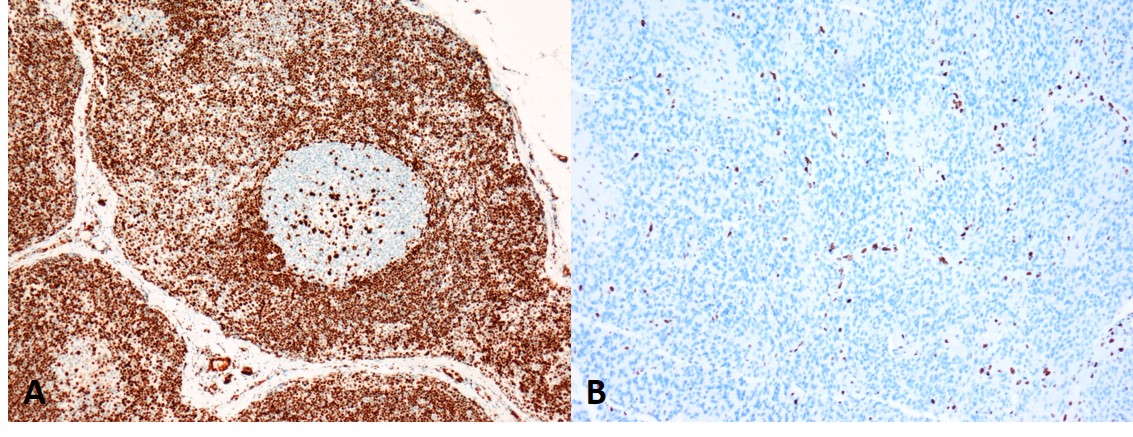


**Supplementary Figure 1:** Canine lymph node showing diffuse immunoreactivity of small lymphocytes for H3K27me3. H3K27me3, 100x (**A**). Human diffuse midline glioma with a histone H3 K27M mutation with immunohistochemical loss of H3K27me3 expression. The positive reaction is visible in the endotelial cells and scattered lymphocytes. H3K27me3, 100x (**B**).
